# Vendors' handling practices of edible long-horned grasshoppers (*Ruspolia differens*) products and implications on microbial safety

**DOI:** 10.3389/fmicb.2024.1385433

**Published:** 2024-05-06

**Authors:** Loretta Mugo-Kamiri, Jasper K. Imungi, Lucy Njue, Gracious Diiro, Fidelis Levi O. Ombura, Komivi S. Akutse, Tanga M. Chrysantus, Fathiya M. Khamis, Sevgan Subramanian

**Affiliations:** ^1^International Centre for Insect Physiology and Ecology (ICIPE), Nairobi, Kenya; ^2^Department of Food Science Nutrition and Technology, Faculty of Agriculture, University of Nairobi, Nairobi, Kenya; ^3^Unit for Environmental Sciences and Management, North-West University, Potchefstroom, South Africa

**Keywords:** edible grasshoppers, *Ruspolia differens*, food safety, microbial contaminants, vendor characteristics

## Abstract

Edible grasshopper, *Ruspolia ruspolia*, has nutritional and cherished cultural and economic importance to people from diverse cultures, particularly in over 20 African countries. It is consumed at home or commercially traded as sautéed, deep-fried, or boiled products. However, there is limited information on the hygiene practices of the vendors and the implications on the microbial safety of the final product. This research aimed at assessing the food safety knowledge, handling practices and shelf life of edible long-horned grasshopper products among vendors and the microbial safety of ready-to-eat products sold in 12 different markets in Uganda. Samples of raw, deep-fried and boiled grasshoppers were randomly collected from 74 vendors (62% street and 38% market vendors) and subjected to microbial analysis. Over 85% of the vendors surveyed had no public health food handler's certificate and >95% had limited post-harvest handling knowledge. Total aerobic bacteria (7.30–10.49 Log10 cfu/g), Enterobacteriaceae (5.53–8.56 Log10 cfu/g), yeasts and molds (4.96–6.01 Log10 cfu/g) total counts were significantly high and above the acceptable Codex Alimentarius Commission and Food Safety Authority of Ireland (FSAI) limits for ready-to-eat food products. Eight key pathogenic bacteria responsible for foodborne diseases were detected and these isolates were characterized as *Bacillus cereus, Hafnia alvei, Serratia marcescens, Staphylococcus aureus, S. xylosus, S. scuiri, S. haemolyticus*, and *Pseudomonas aeruginosa*. Findings from this study highlight the urgent need to create local and national food safety policies for the edible grasshopper “*nsenene*” subsector to regulate and guide street and market vending along the value chain, to prevent the transmission of foodborne diseases to consumers.

## 1 Introduction

Insects are consumed in many countries in Africa, Asia, and South America (Kelemu et al., 2015; van Huis, 2015). Over 2,000 insect species are reportedly consumed globally by more than 3,071 ethnic groups (Ramos-Elorduy, 2009; van Huis, 2015). In Uganda, the edible grasshopper, *Ruspolia differens* Serville locally known as “*nsenene*” is a major delicacy, providing food, nutrition and income to many households (Agea et al., 2008; Leonard et al., 2020, 2022). *Nsenene* is highly nutritious, containing an average of 44% protein and 48% fat, of which 34% is polyunsaturated (Paul et al., 2016). Consuming 100 grams of *nsenene* would contribute significantly to the recommended daily requirements of retinol, α-tocopherol, niacin, riboflavin and folic acid as well as iron, zinc, calcium, and potassium (Kinyuru et al., 2010). It has the potential to be used in the fortification of other food products which lack essential nutrients, and also as a commercial source of lipids and proteins (Paul et al., 2016).

*Nsenene* is harvested from the wild during the swarming seasons of April to June and November to January each year, mainly in Uganda's Central and Western parts. During the swarming seasons, these edible insects create a profitable microenterprise involving their harvesting, processing and marketing by vendors (Ndimubandi et al., 2018; Sengendo et al., 2021). The key players of the *nsenene* value chain include collectors who harvest the insects during the night, transporters, wholesalers, processors, and vendors (Agea et al., 2008; Ndimubandi et al., 2018). Marketing of edible grasshoppers in Uganda is predominantly carried out in the informal markets and along the roads by street food vendors. Like most developing countries, street food vending plays a critical role in Uganda's economy because it provides food that is cheap, readily available and meets the food and nutrition demands of the urban dwellers, while providing the vendors a decent income, sometimes above minimum wage (Muinde and Kuria, 2005; Schuurmans, 2016).

However, street foods tend to be regarded as unsafe and are associated with a greater risk of contamination according to Food Agriculture Organization of the United Nations (2009). Street foods are usually prepared in conditions that expose them to contamination by air, flies, rodents, people, and other foods which are likely to carry harmful bacteria, viruses, parasites and chemicals. In addition, the majority of the street food vendors tend to have limited knowledge, on food hygiene and food safety (Muyanja et al., 2011). According to the World Health Organization, “an estimated 600 million—almost 1 in 10 people in the world fall ill after eating contaminated street food and 420,000 die every year, resulting in the loss of 33 million healthy life years (DALYs)” (Havelaar et al., 2015). Children under 5 years of age carry 40% of the food-borne disease burden, with 125,000 deaths every year. In Uganda, food and water-borne diseases fall under infectious diseases with the highest degree of risk, especially bacterial diarrhea, hepatitis A and E and typhoid fever (US CIA, 2016).

Although there are minimum requirements for processing, packaging, storage, handling, and delivery of foods intended for human consumption, there are no regulations and standards specific to edible insects such as *nsenene* in Uganda. The lack of regulations and standards coupled with the informal nature of the *nsenene* subsector put consumers at great risk of contracting foodborne diseases due to potential contamination along the value chain. Given the economic importance of the edible insect subsector, there is an urgent need to formulate proper standards and regulations to ensure the quality of the edible insect product along the value chain. However, this requires strong evidence-based data to demonstrate the status of microbial safety of marketed *nsenene* as well as the food safety knowledge, and handling practices among vendors in Uganda. Hence, the main objective of this study was to assess whether *nsenene* sold on the streets and marketplaces met the minimum microbial food safety standards of marketed ready-to-eat products. The references used were those set by the Codex Alimentarius Commission (CAC) and the Food and Safety Authority of Ireland (FSAI). The CAC creates standards and guidelines to ensure safe, nutritious food worldwide. These standards cover hygiene practices, additives, contaminants, and microbiological criteria (FAO/WHO Codex Alimentarius Commission, 2007). The Food Safety Authority of Ireland (FSAI) on the other hand sets microbial limits in ready-to-eat foodstuffs, based on scientific research and risk assessment, to ensure public health and quality (Food Safety Authority of Ireland, 2019). The limits are used by stakeholders to minimize foodborne pathogens and contaminants and promote consumer confidence in the food supply chain.

This study further investigated whether the vendors of the popular *nsenene* snacks have basic knowledge of post-harvest handling practices of the edible insects, preservation, and storage measures undertaken as well as sanitation and personal hygiene practices of vendors. Hence, this study was carried out in the Kampala and Masaka districts of Uganda, where outbreaks of *nsenene* swarming were observed. Masaka district was purposively selected for this study since it is the main swarming region, where the grasshoppers are harvested, processed and marketed along the road by street vendors. Masaka town is situated close to the equator in Central Uganda on the West of Lake Victoria, about 140 Km from Kampala. The town had a population of 103,829 in the 2014 Uganda national census and covers an area of 58 Km^2^ (UBOS, 2017). It is the major swarming area for *nsenene* as it is one of the wettest districts in Uganda with an average annual rainfall of 1,174 mm. Similarly, Kampala was selected because it is the main market for *nsenene* collected from swarming areas. Kampala is the national and commercial capital of Uganda. The city covers an area of 181 Km^2^, stands at an elevation of 1,190 m above sea level and has a projected population of 1.65 million in 2019 (Uganda National Bureau of Statistics, 2017).

## 2 Materials and methods

### 2.1 Study sites and season

**Figure 1:**
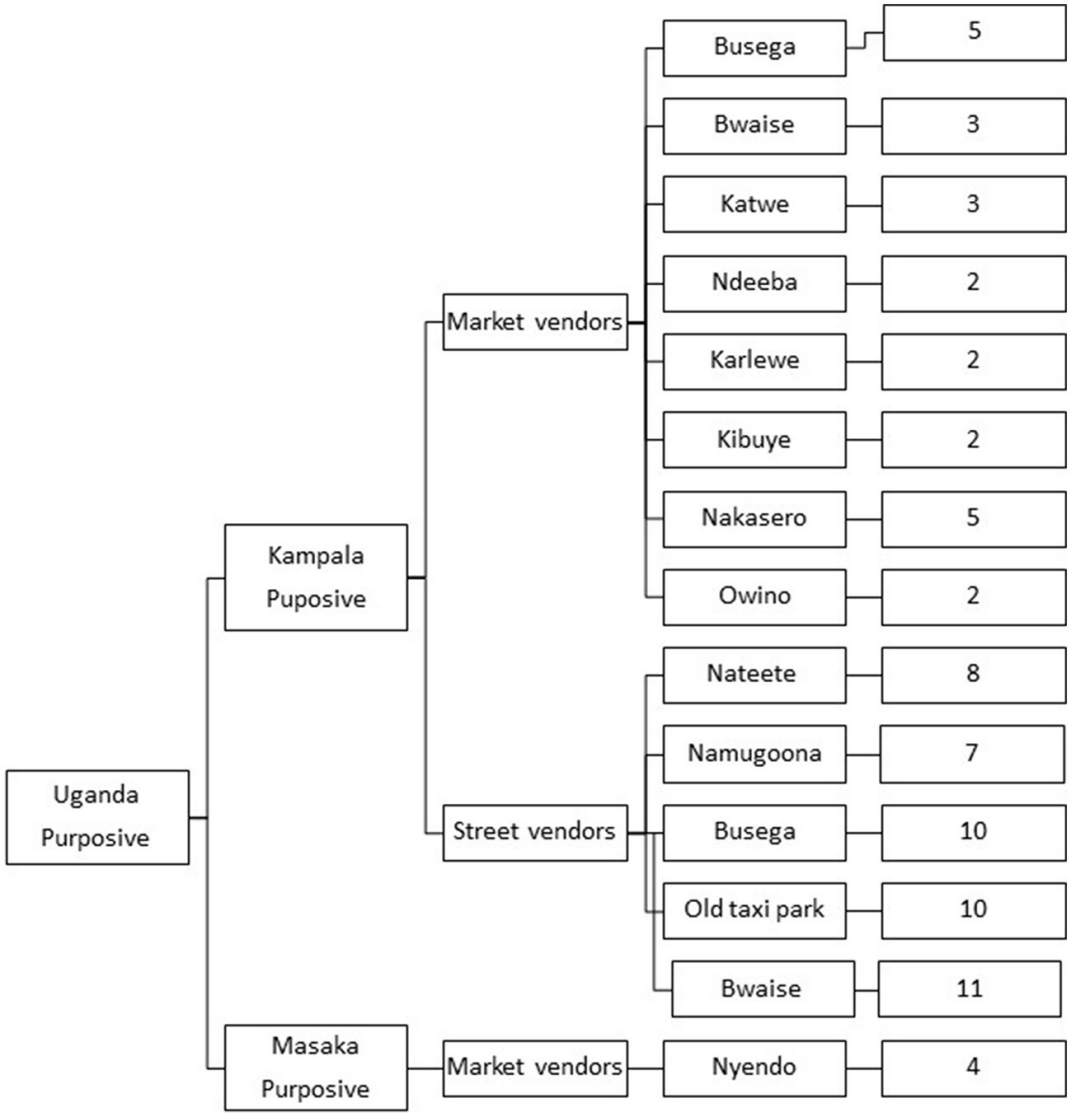
Distribution of vendors in different market locations.

A total of 12 market locations were selected for the study based on the presence of edible grasshopper vendors according to local informant recommendations (Figure 1). These included 11 markets in Kampala (Nakasero, Busega, Bwaise, Old Taxi Park, Ndeeba, Nateete, Katwe, Karlewe, Namugoona, Kibuye, and Owino), and a single market (Ngendo market) in Masaka.

### 2.2 Sample size and sampling procedure

**Figure 2:**
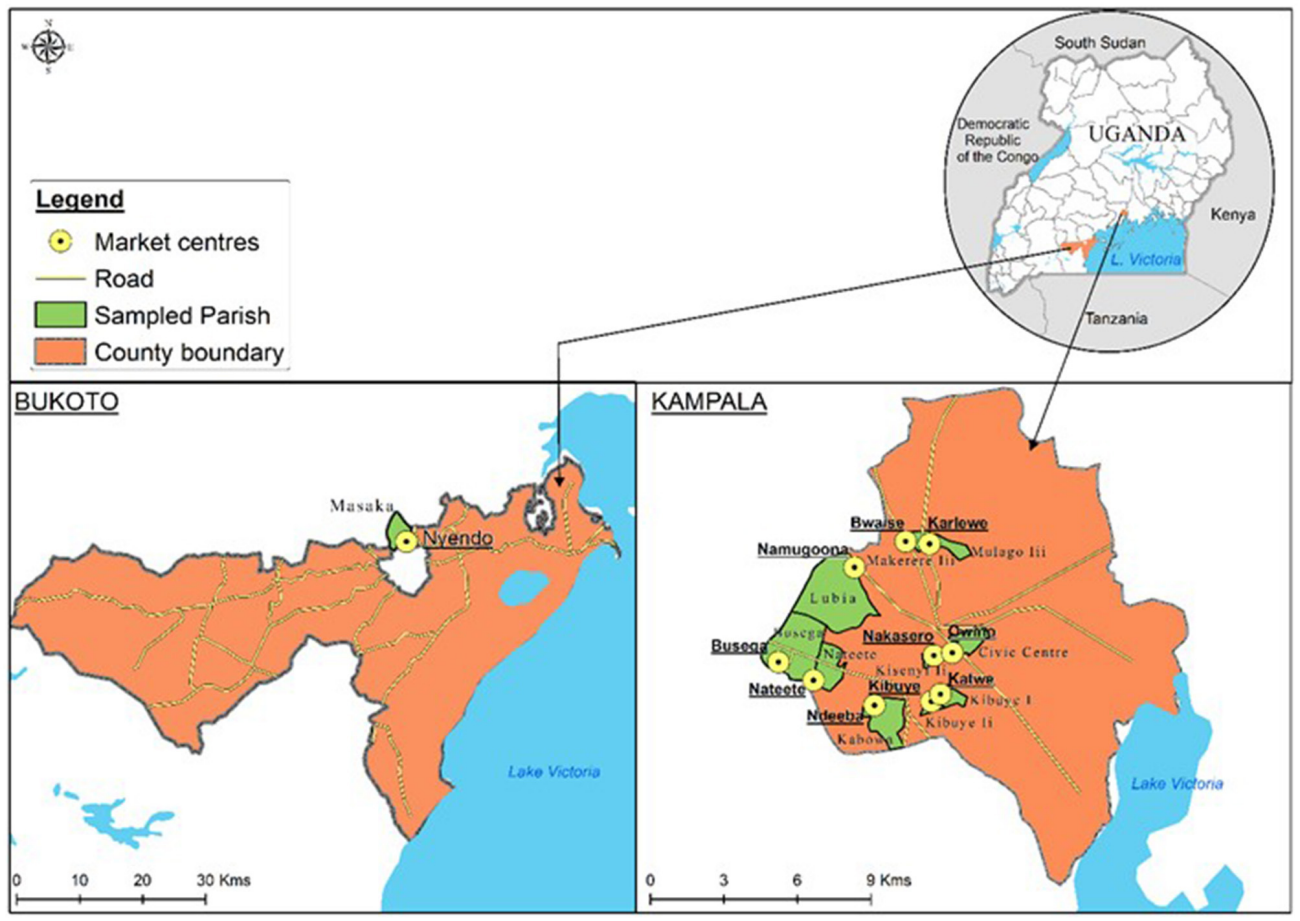
Map showing markets sampled in Kampala and Masaka.

This was a cross-sectional study with analytical and descriptive components. The descriptive component involved an interview-based survey of grasshopper vendors (Supplementary material 1) to assess their knowledge of food safety, personal hygiene and sanitation in insect processing and marketing. A census of all the vendors present was conducted, where a total of 74 vendors (62% street and 38% market vendors), from 12 market locations who processed and sold edible grasshoppers along the streets or in stationary market stalls, were selected for this study as illustrated in Figure 2. The analytical component involved buying *nsenene* (grasshopper) from vendors and assessing microbial contamination by estimating levels of total viable count, *Enterobacteriaceae*, yeasts and mold and investigating the microbial diversity using molecular tools. Grasshoppers sampled were categorized according to the processing method i.e., raw, boiled, and deep fried, storage time, market location, and type of vendors [i.e., market or street vendors].

### 2.3 Collection of insect samples for microbial analyses

During sampling, standard microbiological guidelines and criteria for sampling and evaluation of ready-to-eat foods were followed (Food Standards Agency, 2000; Gilbert et al., 2000; Food Safety Authority of Ireland, 2019). From each market, ~250 g of either fresh, deep-fried or boiled grasshoppers were obtained from each vendor from each location. The samples were packaged in sterile poly bags (SDP Inc, Quebec, Canada) then placed in plastic containers, tightly sealed and transported in cool boxes that had temperature stabilization cool packs (to maintain conditions of 4°C) during transport to the *icipe*'s Arthropod Pathology Unit (APU) laboratories for further analysis. Transport to the APU laboratories was within 24–48 h of sample collections from the respective regions.

### 2.4 Microbial analyses

#### 2.4.1 Culturing of microbes

Sample preparation was performed according to ISO 6887–1:2017 (International Organization for Standardization, 2017) and the estimation of microbial counts was done according to the EU Commission Regulation No 2073/2005 on “Microbiological Criteria for Foodstuffs” (European Union, 2005) about 5 g of the sample was weighed using an analytical scale and homogenized with a pestle and mortar in a mixture with 45 ml of sterile distilled triton water (0.05%). A 10-fold serial dilution series was done up to 10^−6^ from which microorganisms' total viable count, *Enterobacteriaceae*, yeast and molds were enumerated. Total viable count (TVC) was enumerated using Luria Bertani (LB) media which contained 10 g tryptone (Oxoid Ltd, Hampshire, UK), 5 g yeast extract agar (Oxoid Ltd), 5 g NaCl (Sigma-Aldrich, Inc, Missouri, USA), and 15 g agar (Oxoid Ltd) per liter of media. These were incubated at 35 ± 2°C for 24 h in a MIR-554 cooled incubator (PHC Europe B.V., Nijverheidsweg, The Netherlands). *Enterobacteriaceae* counts were enumerated using Violet red bile glucose agar (Oxoid Ltd), incubated at 37°C for 24 h in a MIR-554 cooled incubator (PHC Europe B.V). Yeasts and molds were determined using Potato Dextrose Agar (Oxoid Ltd) incubated at 25°C for 5 days in a MIR-554 cooled incubator (PHC Europe B.V). Using the spread plate technique, 100 microliters of sample was spread with a sterile spreader, over media that had solidified on a Petri dish and incubated in the respective temperatures. Colony forming units were counted when the colonies were between 30 and 300 then the data was converted to log10 cfu/g of sample using the formula Log CFU/mL = Log10 [CFU/(dilution factor x aliquot volume)] in MS Excel.

#### 2.4.2 Molecular characterization of bacteria, fungi, yeasts, and molds isolates

##### 2.4.2.1 Pure culture isolations and genomic DNA extractions

Pure bacterial and fungal isolation was carried out according to the methods described by Gatheru et al. (2019). From each pure bacterial isolate plate, individual bacterial colonies were picked and then grown in Nutrient Broth (Oxoid, UK) for 24 h at 32 ± 2°C in a MIR-554 cooled incubator (PHC Europe B.V). From this bacterial broth, 1.5 mL was collected, and the bacterial cells were harvested through centrifugation (5427R centrifuge, Eppendorf, Hamburg, Germany) at 8,000 rcf for 5 min at 4°C. Genomic bacterial DNA extraction was done using the Isolate II genomic DNA extraction kit (Meridian Bioscience, London, UK) as per the manufacturer's instructions. On the other hand, fungi spores/mycelia were scrapped off from pure fungal culture plates. The spores/mycelia were collected in 2 mL Eppendorf tubes and genomic fungal DNA was extracted using the Isolate II Plant DNA Extraction Kit (Meridian Bioscience) following the manufacturer's instructions. The extracted bacteria and fungi DNA was quantified using a NanoDrop 2000 Spectrophotometer (Thermo Fisher Scientific, Wilmington, USA). Bacteria and fungi DNA samples were stored at −20°C awaiting Polymerase Chain Reaction (PCR) analysis.

##### 2.4.2.2 Polymerase Chain Reaction, purification and sequencing

The bacterial 16S rRNA gene region was amplified using 27F (5′-AGAGTTTGATCMTGGCTCAG-3′) and 1492R (5′-TACCTTGTTACGACTT-3′) primers (Lane et al., 1985). PCR amplification was done in total reaction volumes of 20 μL containing 5X My *Taq* Reaction Buffer (comprising of 5 mM dNTPs, 15 mM MgCl_2_, stabilizers and enhancers), 0.5 pmol μL^−1^ of each primer, 0.5 mM MgCl_2_, 0.0625 U μL^−1^ My *Taq* DNA polymerase (Meridian Bioscience), and 15 ng μL^−1^ of DNA template then set up in the Eppendorf Mastercycler^®^ Nexus Gradient Thermal Cycler (Eppendorf, Hamburg, Germany) using the following conditions: initial denaturation at 95°C for 2 min followed by 40 cycles of denaturation at 95°C for 30 s, annealing at and primer elongation at 72°C for 1 min. The final extension step lasted for 10 min at 72°C. The expected product size ranged between 1,450 and 1,500 bp. On the other hand, the fungi intergenic transcribed spacer region (ITS) was targeted through PCR using ITS4 (5′-GGAAGTAAAAGTCGTAACAAGG-3′) and ITS5 (5′-TCCTCCGCTTATTGATATGC-3′) primers (White et al., 1990). The PCR reaction set-up was as described above except for an annealing temperature of 59°C for 40 s. The expected product size ranged between 400 and 450 bp.

The amplified PCR products were resolved through a 1.2% agarose gel, then the DNA bands on the gel were visualized and documented using the KETA GL imaging system trans-illuminator (Wealtec Corp, Meadowvale Way Sparks, Nevada, USA). The bands were later excised and then purified using Isolate II PCR and Gel Kit (Meridian Bioscience) following the manufacturer's instructions and shipped to Macrogen Europe BV (Meibergreef, Amsterdam, the Netherlands), for bi-directional sequencing. The resultant sequences obtained were assembled and edited using BioEdit software version 7 (Hall, 1999). For conclusive identification of the species, similarity searches were conducted by querying the consensus sequences via the BLASTn (Basic Local Alignment Search Tool) algorithm at the GenBank database [hosted by the National Center of Biotechnology Information (NCBI)]. Visualization of the characterized isolates was done in a chord diagram run in R Studio (v. 4.1.0; R Core Team, 2020).

### 2.5 Market survey

A semi-structured, pre-tested questionnaire (Supplementary material 1) was used as the data collection tool in the descriptive study. The questionnaire was divided into three sections, the first focused on the socio-demographic characteristics of the vendors, i.e., age, gender, level of education length of time in the grasshopper vending business and the ownership of the business. The second section contained specific questions to test the respondents' knowledge of food safety such as quality and hygiene factors considered when purchasing raw grasshoppers, cleaning, sorting of grasshoppers, preservation, storage and frequency of changing deep frying oil as well as their knowledge on foodborne illnesses. The third section was an observation checklist to score the personal and food hygiene and sanitation practices of vendors and their working environment. The food hygiene and practice score was an index between 0 and 1 constructed from 13 indicator variables in the checklist. For every good practice, a score of 1 was assigned while 0 was given for poor practice.

### 2.6 Statistical analysis

Descriptive data was analyzed using Stata statistical software (StataCorp, 2013). Descriptive statistics were used to obtain means and standard deviations. A significance of ≤ 0.1 was used for the data on the survey. For the analytical component, the data on TVC, *Enterobacteriaceae* and fungi were analyzed using analysis of covariance where the storage time was the covariate, and the preparing method was the treatment. Least squares means were estimated for each treatment using the *emmeans* package of R software version 3.5.3 (R Core Team, 2019). The least squares means were separated using the adjusted Tukey method.

## 3 Results and discussion

### 3.1 Microbial load of marketed edible grasshoppers (*nsenene*)

The mean bacterial population of the samples analyzed are presented in Table 1. The *Enterobacteriaceae*, TVC, and yeasts and molds loads recorded were above 5.0 log cfu/g, except for the fungal load from deep-fried samples collected from market vendors which was borderline (4.96 ± 0.33 log cfu/g). These results suggest high levels of contamination beyond acceptable limits for ready-to-eat foods (Food Safety Authority of Ireland, 2019). Furthermore, the microbial counts obtained were benchmarked against the general principles of food hygiene and process hygiene guidelines included in the Codex Alimentarius Commission (CAC) similar to other studies related to insect microbial contamination (Stoops et al., 2016). Per the CAC guidelines, both *Enterobacteriaceae* and total viable counts, which are used as process hygiene indicators exceeded the recommended level, which is 3 log10 cfu/g for *Enterobacteriaceae* and 5 log10 cfu/g for TVC for minced meat and other ready-to-eat foods (Food Safety Authority of Ireland, 2019). Similar high levels of contaminants have been reported for fresh *nsenene* in Uganda with levels of TVC ranging from 8.38 to 9.41 log10 cfu/g, *Enterobacteriaceae* from 6.89 to 7.83 log10 cfu/g, and yeast and molds counts 5.77 to 7.12 log10 cfu/g (Ssepuuya et al., 2019). Other insects such as the African migratory locust, *Locusta migratoria migratorioides* had TVC, *Enterobacteriaceae*, yeasts and mold levels ranging between 7.8–8.6; 7.1–7.6, and 5.0–5.4 log10 cfu/g, respectively (Stoops et al., 2016). Fresh mealworms and crickets with TVC values ranging between 6.7 and 7.7 log cfu/g and *Enterobacteriaceae* between 4.2 and 6.8 log10 cfu/g have been reported (Klunder et al., 2012). The level of *Enterobacteriaceae* among fresh, deep-fried, and boiled grasshopper samples varied significantly (*P* < 0.05; Table 1). There was a weak positive correlation between TVC load and the storage time of the samples (*r* = 0.37, *P* = 0.008). However, the storage time did not significantly influence the *Enterobacteriaceae* load, yeast, and molds loads (*r* = 0.008, *P* = 0.96) implying that most of the contaminants might have been due to poor food handling practices rather than storage time.

**Table 1:** Mean (± S.E.) estimates of microbial contamination of marketed grasshoppers (*nsenene*).

**Processing method**	**Type of vendor**	**TVC^†^**	**Enterobacteriaceae^†^**	**Yeast and mold^†^**
Fresh	Market vendors	8.61 ± 0.51	8.56 ± 0.44^b^	6.01 ± 0.43
Deep fried		7.30 ± 0.28	6.51 ± 0.27^a^	5.79 ± 0.25
Boiled		8.51 ± 0.98	5.74 ± 0.84^a^	5.77 ± 0.84
		*F*_2, 30_ = 2.73, *P* = 0.816	*F*_2, 26_ = 8.83, *P* = 0.001	*F*_2, 30_ = 0.098, *P* = 0.907
Deep fried	Street vendors	8.17 ± 0.38	5.53 ± 0.93	4.96 ± 0.33
Boiled		10.49 ± 1.11	6.80 ± 0.32	6.59 ± 0.95
		*F*_1, 13_ = 3.703, *P* = 0.0765	*F*_1, 13_ = 1.594, *P* = 0.228	*F*_1, 13_ = 2.530, *P* = 0.136

^†^Mean ± SD of microbial contamination expressed as Log_10_ cfu g^−1^.

^a, b^Mean values on the same column, followed by different letters, indicate that they differ significantly (p < 0.05).

In general, such a high microbial count as observed in this study indicates the potential presence of food-borne pathogens and toxins that can be harmful to consumers (Food Safety Authority of Ireland, 2019). Such contaminant levels can be attributed to poor hygiene and sanitation during harvesting and processing, fecal contamination, inappropriate storage, and inadequate cooking.

### 3.2 Molecular characterization of bacterial isolates from *Ruspolia differens*

**Figure 3:**
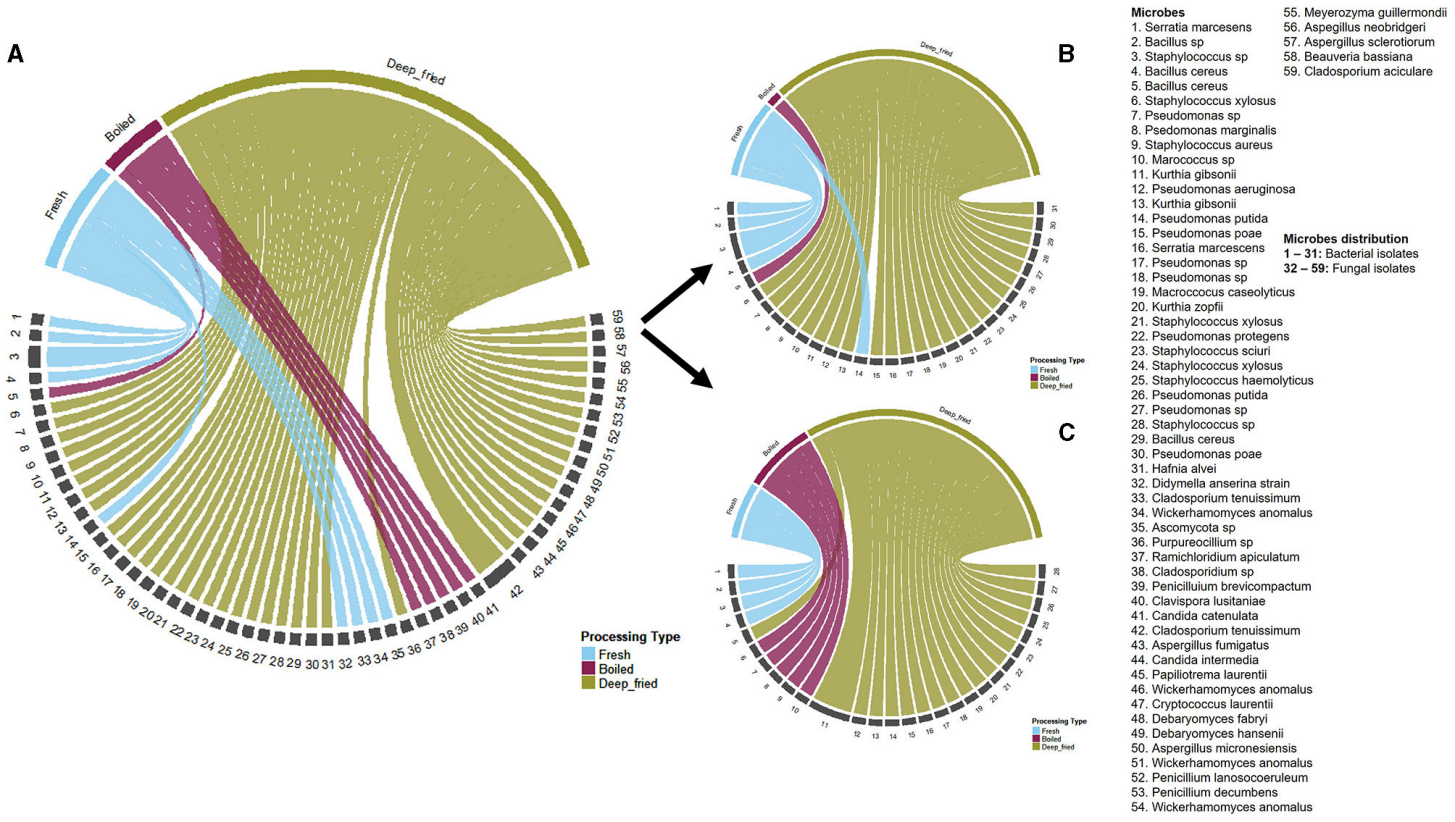
A chord diagram illustration of the microbes isolated from fresh, boiled, and deep-fried edible *nsenene* (grasshoppers) from different market locations. **(A)** Schematic illustration of all the microbes isolated from fresh, boiled, and deep-fried *nsenene*; **(B)** All the bacteria isolated from fresh, boiled, and deep-fried *nsenene*; **(C)** All fungi isolated from fresh, boiled, and deep-fried *nsenene*.

Figure 3A represents a schematic illustration of all the microbes isolated from fresh, boiled, and deep-fried grasshopper (*nsenene*) products. A total of 31 bacterial isolates (both gram-positive and gram-negative) were isolated and characterized (Figure 3B), out of which eight key pathogenic bacteria were identified. These pathogenic bacteria included *Bacillus cereus, Hafnia alvei, Serratia marcescens, Staphylococcus aureus, S. xylosus, S. scuiri, S. haemolyticus*, and *Pseudomonas aeruginosa*. *Bacillus cereus*, a gram-positive bacteria adapted to grow in the gut of insects and mammals (Stenfors Arnesen et al., 2008) was isolated from deep-fried grasshoppers that had been stored for 21 days. *Bacillus cereus* is known to cause emetic food poisoning and bloody diarrhea (Guinebretière et al., 2002).

From the family *Enterobacteriaceae*, two major bacteria species *Hafnia alvei* Møller and *Serratia marcescens* were isolated. *Hafnia alvei* was isolated from deep-fried grasshoppers that had been stored for 30 days while *Serratia marcescens* was isolated fresh un-processed and deep-fried grasshoppers that had been preserved for 1 day. *Serratia marcescens* is classified as a class I pathogen of both grasshoppers and humans (Grabowski and Klein, 2017; Leonard et al., 2023). In humans, *S. marcescens* has been reported to cause nosocomial infections (Castelli et al., 2008) but in grasshoppers, no epizootics have been reported, although its presence signifies that it can be a potential pathogen in a mass-rearing environment.

In the family *Micrococcaceae*, four bacteria strains: *Staphylococcus aureus, Staphylococcus xylosus, Staphylococcus sciuri* subsp. sciuri., and *Staphylococcus haemolyticus* were isolated. *Staphylococcus aureus* (isolated from deep-fried grasshoppers) is a natural part of the natural human flora but can cause Staphylococcal Food Poisoning (SFD) in some conditions (Lowy, 1998; Hennekinne et al., 2012; Taylor and Unakal, 2019). *Staphylococcus aureus* is transmitted through improper food handling (i.e., direct contact or sneezing and coughing into food by handlers). *Staphylococcus sciuri* (isolated from deep-fried grasshoppers stored for 10 days) and *S. xylosus* (isolated from deep-fried grasshoppers stored for 0, 4, and 21 days) are generally non-pathogenic, but some strains have been reported to carry an enterotoxin gene (Rodríguez et al., 1996; Udo et al., 1999). *Staphylococcus sciuri* has previously been reported from fresh-winged *R. differens* in Uganda (Ssepuuya et al., 2019). *Staphylococcus haemolyticus* (isolated from deep-fried grasshoppers stored for 21 days) is however highly pathogenic causing septicemia, peritonitis, otitis, and urinary tract infections with reports of high levels of antibiotic resistance (Takeuchi et al., 2005).

*Pseudomonas aeruginosa* (isolated from deep-fried grasshoppers) from the family *Pseudomonadaceae* is one of the top three causes of opportunistic human infections (Stover et al., 2000). *Pseudomonas aeruginosa* frequently occurs in wounds (Hardalo and Edberg, 1997), therefore insect-based food handlers with wounds can be carriers of this bacteria, which can be transmitted through improper food handling. Some spoilage bacteria such as *Kurthia gibsonii* were isolated from grasshopper samples and are widely known to be responsible for meat spoilage. Other species such as *P. protegens* and *P. putida* are known plant growth promoters, while *P. marginalis* are plant pathogens possibly transmitted to the grasshoppers through contact with vegetation materials.

### 3.3 Characterization of fungi, yeasts, and mold species from *Ruspolia differens*

A total of 28 isolates of fungi, yeasts and mold species were characterized from the grasshopper samples (Figure 3C). Several mycotoxins-producing *Aspergillus* species were identified including *Aspergillus fumigatus* and *A. neobridgeri. Aspergillus fumigatus*. These produce mycotoxins such as verruculogen, fumitremorgin A, B and C, fumigaclavines A, B and C and gliotoxin that can be fatal in immunocompromised individuals (Land et al., 1987; Bauer et al., 1989; Lin et al., 2001). *Aspergillus neobridgeri* produces penicillic acid and Xanthomegnis mycotoxins which could have a synergistic toxic effect (Frisvad et al., 2004).

*Penicillium brevicompactum* was one of the toxicogenic fungi isolated from boiled grasshopper samples, commonly associated with fruits and root crops (Pitt, 2006). *P. brevicompactum* can produce mycotoxin known as mycophenolic acid with potent immunosuppressant properties (Overy and Frisvad, 2005), which needs to be further investigated with human consumption of edible grasshoppers.

Yeast-like fungus, *Meyerozyma guillermondii* isolated from deep-fried grasshoppers is known to be an opportunistic pathogen in humans and animals responsible for cutaneous infections in immunosuppressed individuals (Molnár et al., 2008; Rivera et al., 2009; Corte et al., 2015). *Papiliotrema laurentii* (*Cryptococcus laurentii*), a yeast species isolated from deep-fried grasshopper samples has been implicated in human infections and has been reported to occur in soil and products contaminated with pigeon excreta (Haider et al., 2013) implying that its presence in grasshoppers could be due to exposure to dust, and soil. Other fungi and yeast isolated from the grasshoppers with the potential to cause human infections in immunocompromized individuals were *Penicillium decumbens* (fungi) and *Rhodotorula mucilaginosa* (yeast; Alvarez, 1990; Nagahama et al., 2006; Tournas et al., 2006; Wirth and Goldani, 2012). *Candida* species (yeast) isolated from boiled grasshoppers included *Clavispora lusitaniae* (*Candida lusitaniae*) and *Candida catenulata*, while *Candida intermedia* yeast was isolated from deep-fried samples. *Candida* species are known to be the most common cause of human fungal infections (Rajkowska and Kunicka-Styczyńska, 2018) such as candidiasis (including septicemia and pyelonephritis) and thus can be a food safety risk in the consumption of grasshoppers.

### 3.4 Socio-demographic characteristics of the vendors

**Figure 4:**
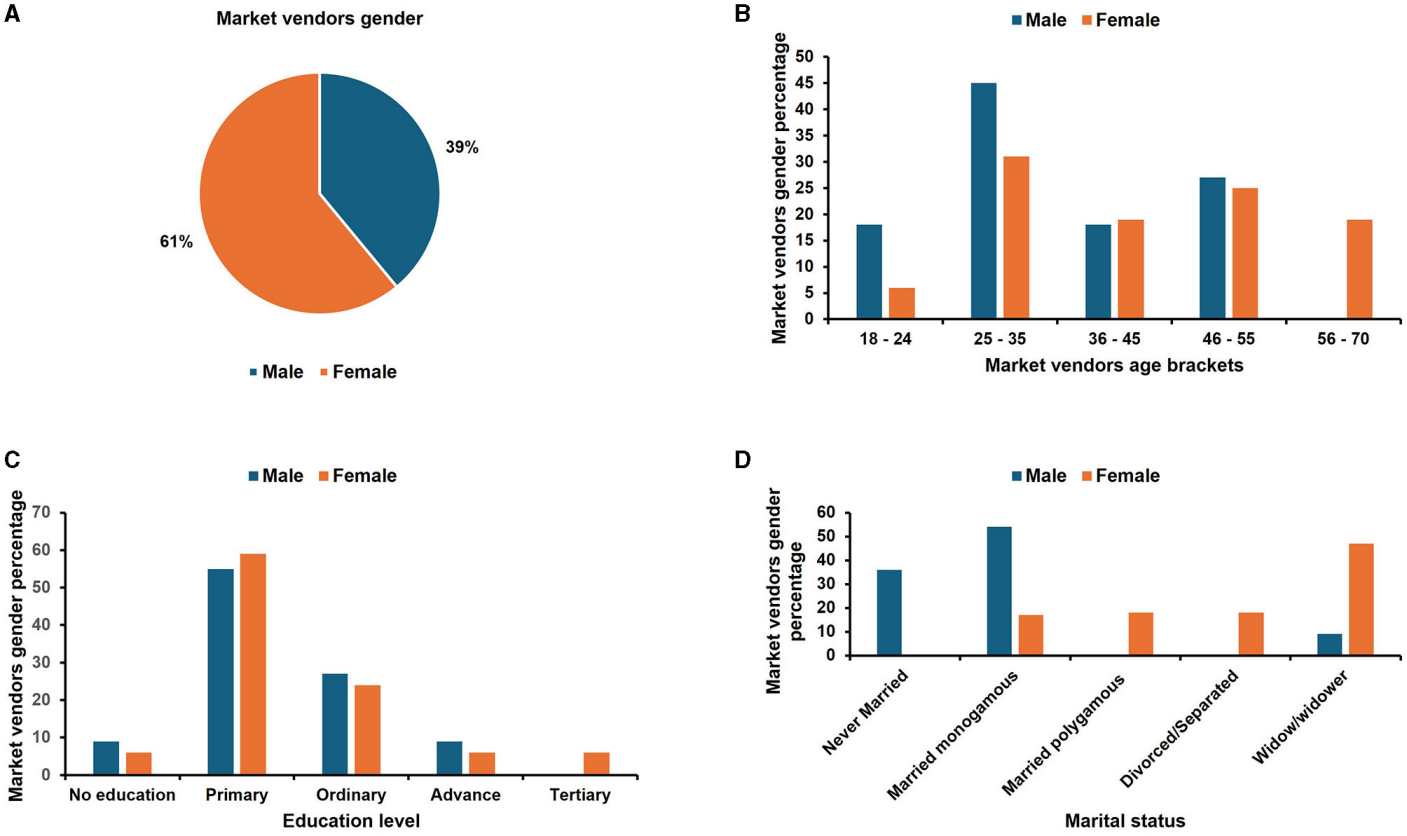
Socio-demographic characteristics of the *nsenene* (grasshopper) market vendors. **(A)** Gender distribution of the market vendors; **(B)** Age brackets of the market vendors; **(C)** education level of the market vendors and **(D)** marital status of the market vendors.

**Figure 5:**
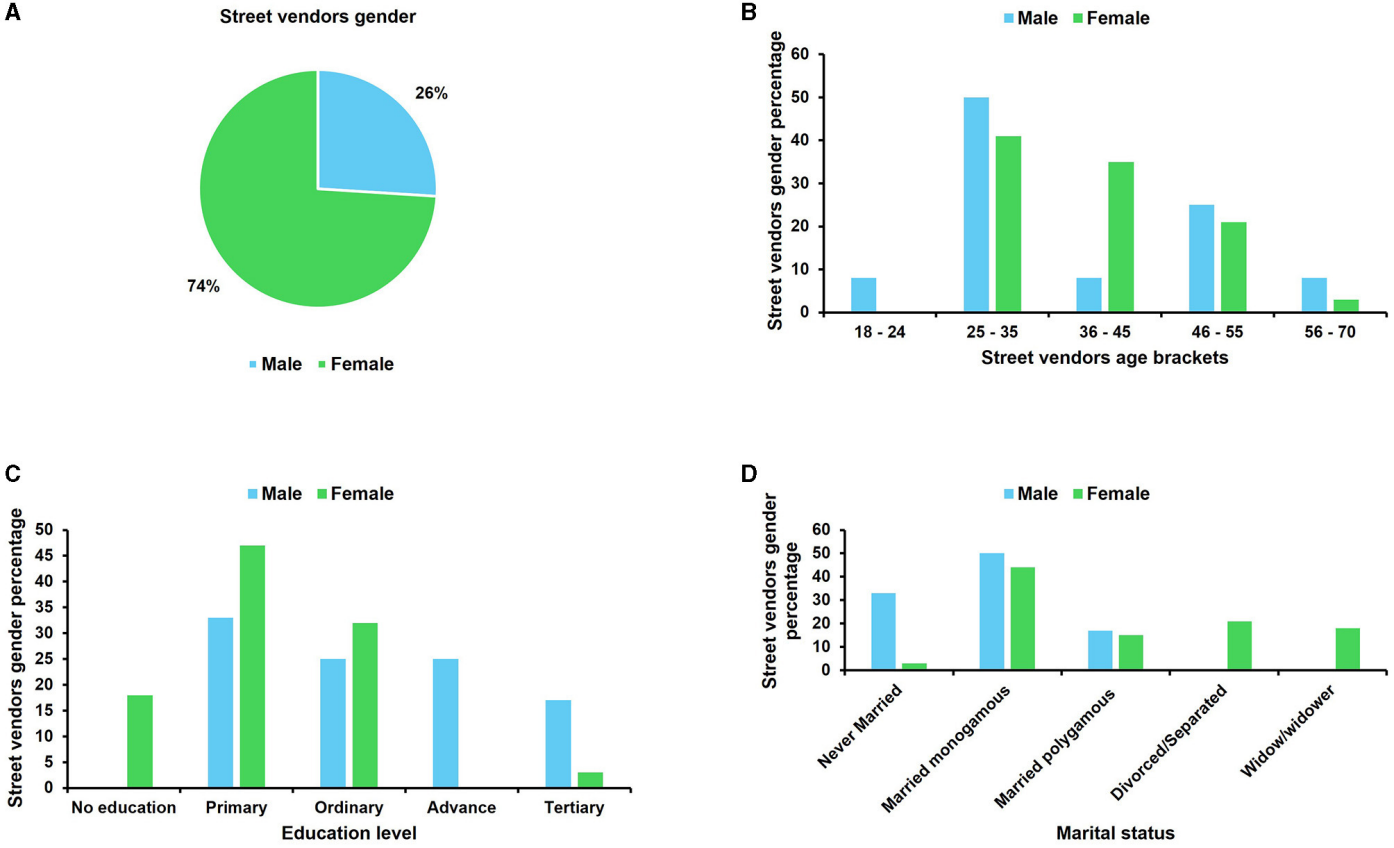
Socio-demographic characteristics of the *nsenene* (grasshopper) street vendors. **(A)** Gender distribution of the street vendors; **(B)** age brackets of the street vendors; **(C)** education level of the street vendors and **(D)** marital status of the street vendors.

Socio-demographic characteristics of the vendors are presented in Figures 4, 5. The results show that most of the vendors were mobile street vendors (SV) (62%) of which 74% were females (Figure 5A). The stationary market vendors (MV) were only 38%, with women accounting for 61% (Figure 4A). Majority of the vendors were between the ages of 25–35 years with males making up a bigger proportion in both categories of market and street vendors (45 and 50%, respectively; Figures 4B, 5B). This suggests greater participation of male youths in the insect-based food enterprise. The majority of both market and street vendors (57 and 43%, respectively) had only primary school level education with female vendors having a greater proportion with primary school education (Figures 4C, 5C). Several studies carried out in Uganda (Muyanja et al., 2011), in Philippines (Alamo-Tonelada et al., 2018) and in Nigeria (Andy et al., 2015) support these observations, which demonstrated similar demographic attributes of street food vendors. Low levels of education of vendors can be associated with poor knowledge of food handling practices which are likely to increase the occurrence of food contamination and foodborne illnesses.

### 3.5 Ownership of the business

**Figure 6:**
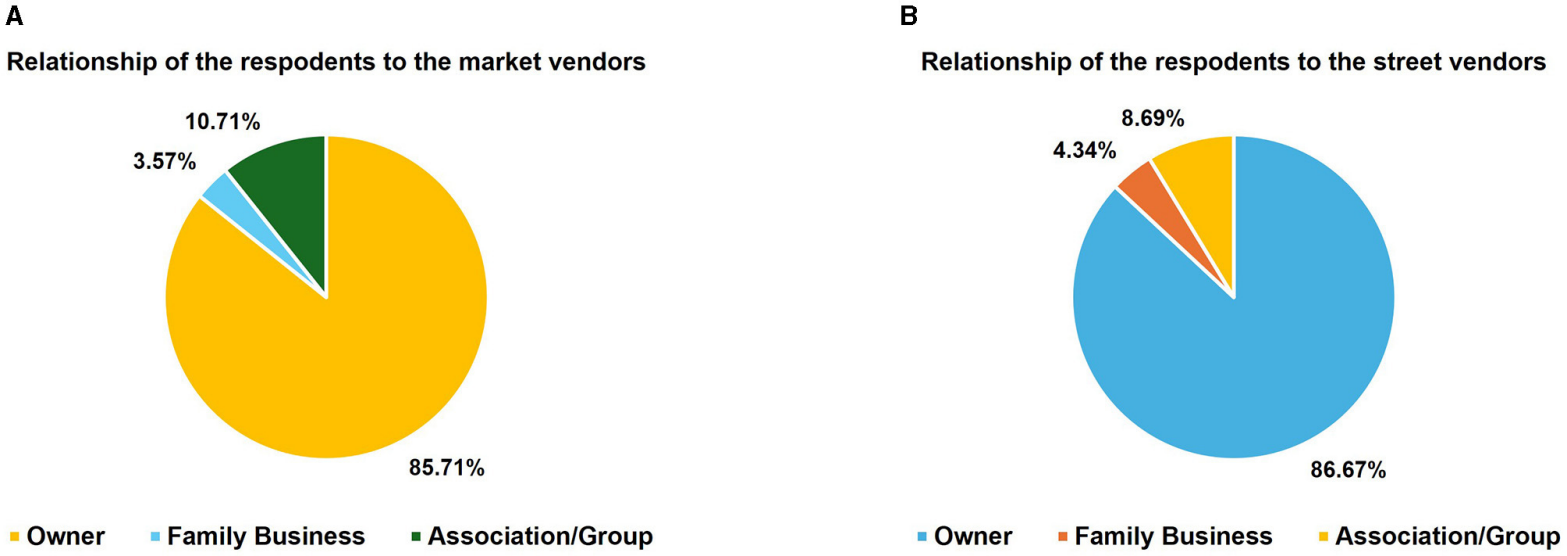
Relationship of the respondents to the *nsenene* (grasshopper) business for **(A)** market vendors and **(B)** street vendors.

**Figure 7:**
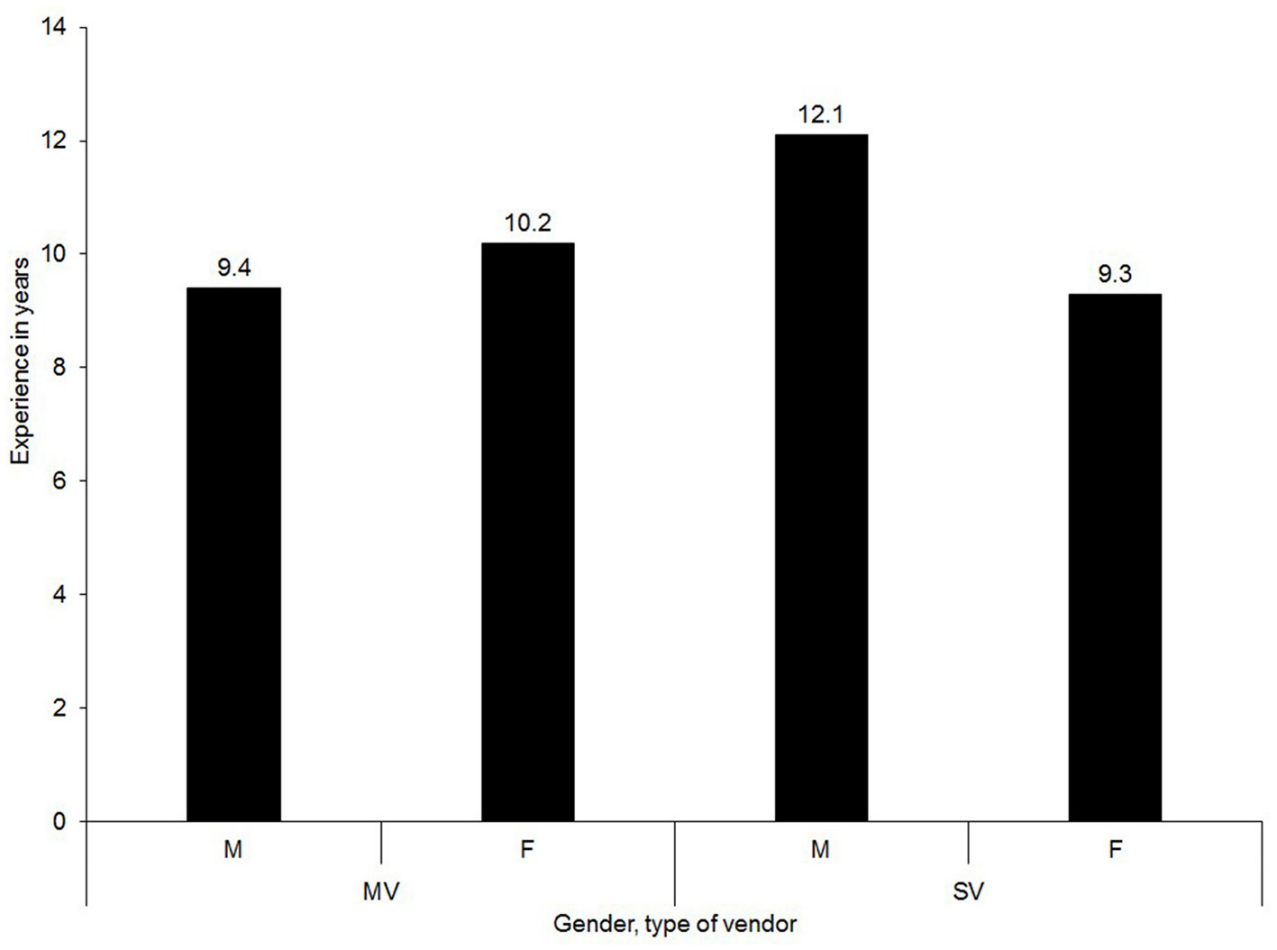
Level of experience of the vendors in the *nesnene* (grasshopper) business.

The relationship of the respondents to the business is detailed in Figure 6 while the level of experience of the vendors in the *nsenene* business is outlined in Figure 7. Majority of the respondents (86% MV and 85% SV) were owners of their grasshopper business with long experiences (9 and 12 years, respectively among market and street vendors). This suggested that grasshopper vending has been a permanent source of income and a longstanding employment option for the youth, especially women. Relative to male vendors, females were more engaged in market vending for a longer period than in street vending (Figure 7). Unlike market vending, street vending tends to be insecure and restrictive, thus a deterrent for women (Bhowmik and Saha, 2012).

### 3.6 Diversity of grasshopper products sold

**Figure 8:**
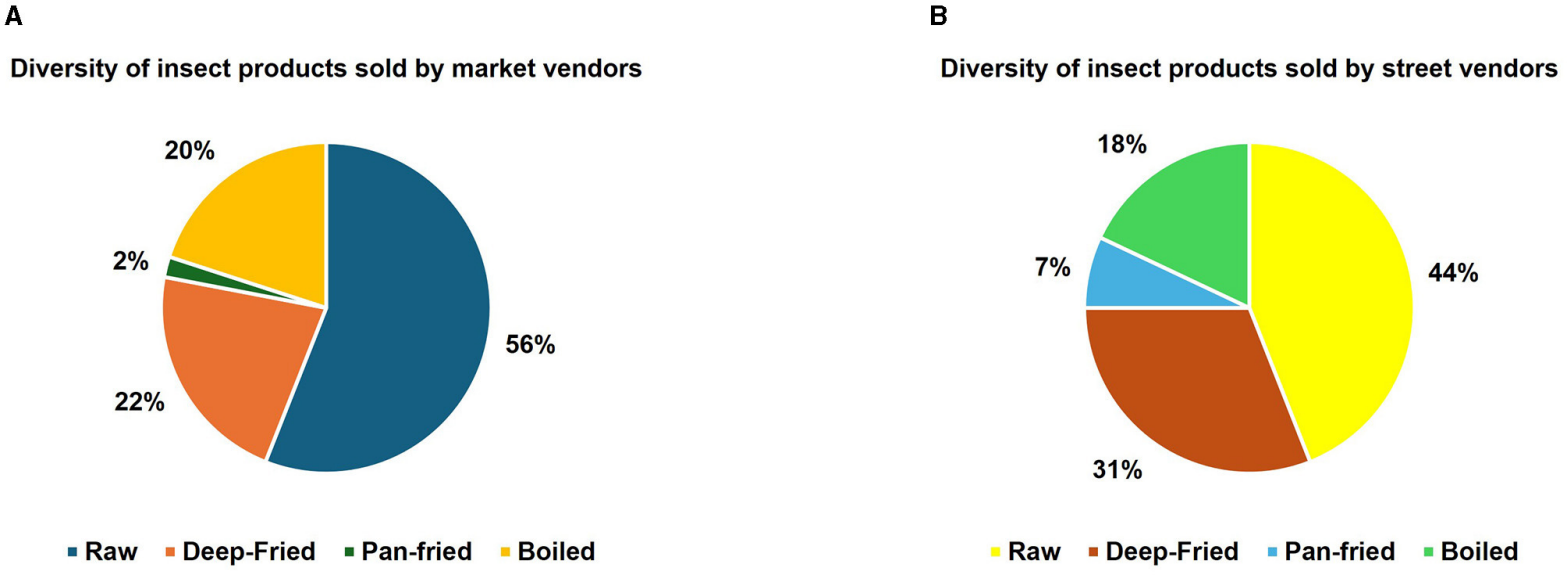
Diversity of *nsenene* (grasshopper) products sold by **(A)** market and **(B)** street vendors.

The results on the vendors' most preferred processing technique of edible grasshoppers are presented in Figure 8. The information obtained indicated that the majority of both street and market (Figures 8A, B) vendors sold raw, unprocessed *nsenene*. Most vendors (>90%), however, claim that unprocessed grasshoppers have a short shelf life of < 24 h hence they fry them to prolong the shelf life rendering deep-fried grasshoppers the second most traded product (Figure 8). Deep-fried grasshoppers were also the most widely available throughout the season possibly due to prolonged shelf life. These results are in line with studies done in Tanzania and Uganda whereby deep-fried grasshoppers are the most common and most preferred, particularly among younger consumers (Biryomumaisho, 2012; Mmari et al., 2017). Boiled grasshoppers are also present in the market, but these also have a short shelf life of fewer than 24 h, hence have to be sold the same day or deep-fried or sun-dried to preserve them further (Figure 8). These findings are in line with other studies conducted in Uganda on the marketing and shelf life of *R. differens* (Ssepuuya et al., 2016; Ndimubandi et al., 2018). Pan-fried grasshoppers tend to be the least popular in Uganda but remain popular in other grasshopper-consuming regions such as Tanzania. In Tanzania, toasting or pan-frying is common because it uses less oil which is seen as a more nutritious and cheaper practice (Mmari et al., 2017).

### 3.7 Health and safety compliance of vendors

#### 3.7.1 Possession of food handler's certificate

Of all the people interviewed, only 5% of the vendors had food handlers' certificates. The Kampala Capital City Authority (KCCA) is the body in charge of enforcing the Public Health Act and monitoring food hygiene practices among food handlers. However, this authority only follows up on food hygiene practices of formally registered food businesses such as hotels, bars and restaurants (KCCA, 2019). This shows a gap in the regulation of informal vending businesses, which represent over 80% of ready-to-eat foods, sold in the informal markets (Roesel and Grace, 2015). These are often left unregulated, and vendors are not made aware of good food handling practices, thus exposing many consumers to the risk of foodborne illnesses.

### 3.8 Food safety knowledge among vendors

#### 3.8.1 Quality attributes considered by vendors when purchasing raw grasshoppers

**Figure 9:**
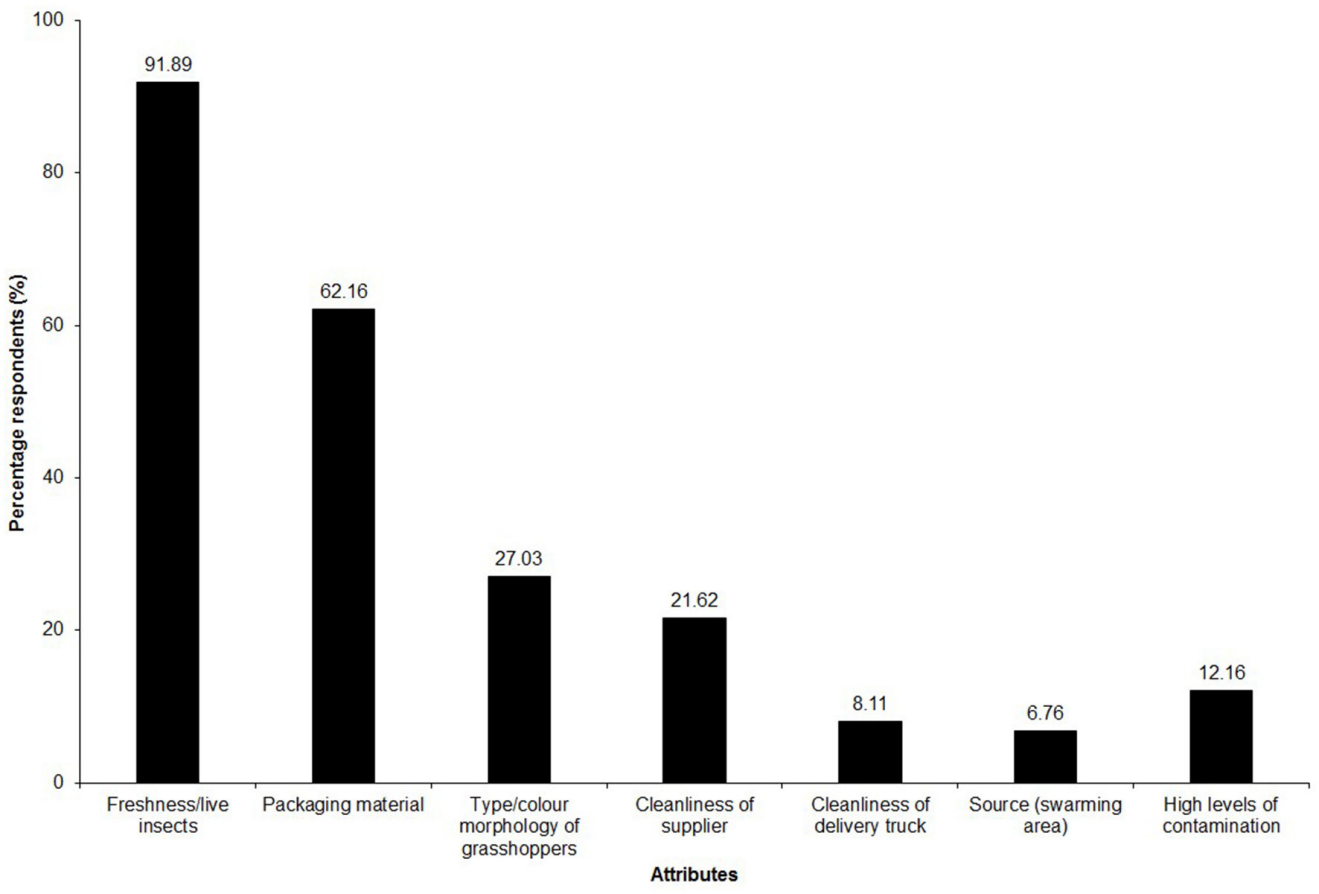
Attributes considered by vendors when purchasing raw *nsenene* (grasshoppers).

The majority of the vendors (>90%) interviewed were keen to buy insects that were still alive despite the higher cost involved compared to dead insects, which were mostly considered to be in bad condition and less fit for consumption (Figure 9). Other attributes considered include clean and well-aerated packaging (reported by 62% of the vendors), and the cleanliness of the collectors and cleanliness of the delivery van (by 41%). Vendors appear to pay attention to the cleanliness of *nsenene* as they sort to remove most of the dirt, dust and other insects caught together with the grasshoppers. However, vendors may not be able to control the contaminants introduced during collection and transportation. For instance, collectors smear grease and oils that may not necessarily be edible, in trapping drums, to prevent insects from escaping thus contaminating the grasshoppers. It is therefore prudent to ensure that raw grasshoppers are handled in the most hygienic way possible before they reach the market to ensure that the final product is of good quality and safe to eat.

#### 3.8.2 Sorting of raw grasshoppers

**Figure 10:**
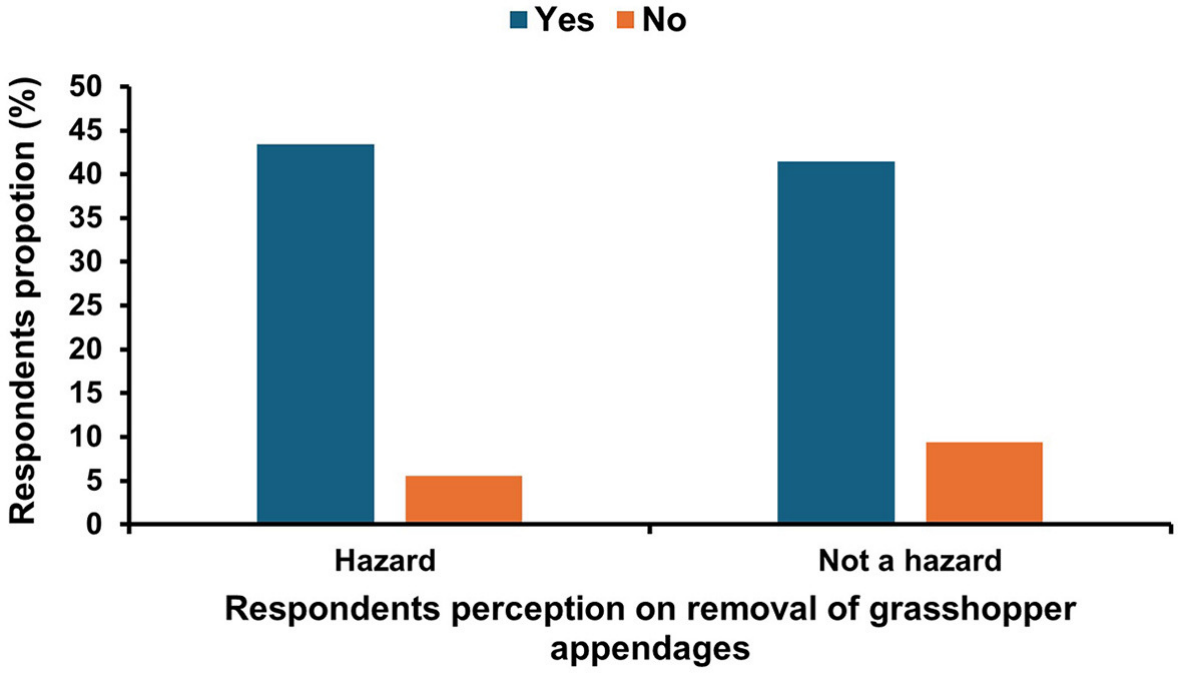
Respondents' perception of *nsenene* (grasshopper) appendages as a hazard.

During our study, it was observed that a key first step in grasshopper processing is the removal of appendages which are the legs, wings and, ovipositor because they are likely to cause harm to the consumers. We, therefore, sought to find out if the vendors understood the practice as one that promoted food safety, and the results are presented in Figure 10. Although more than 80% of the vendors indicated that they removed the grasshopper appendages, only 43.4% of them knew that they are a physical hazard to consumers. They noted that if not removed, appendages may cause choking and constipation, especially in children. About 9.4% of the vendors did not remove the appendages and did not know they were hazardous.

#### 3.8.3 Vendors knowledge of safe use of deep-frying oil

**Figure 11:**
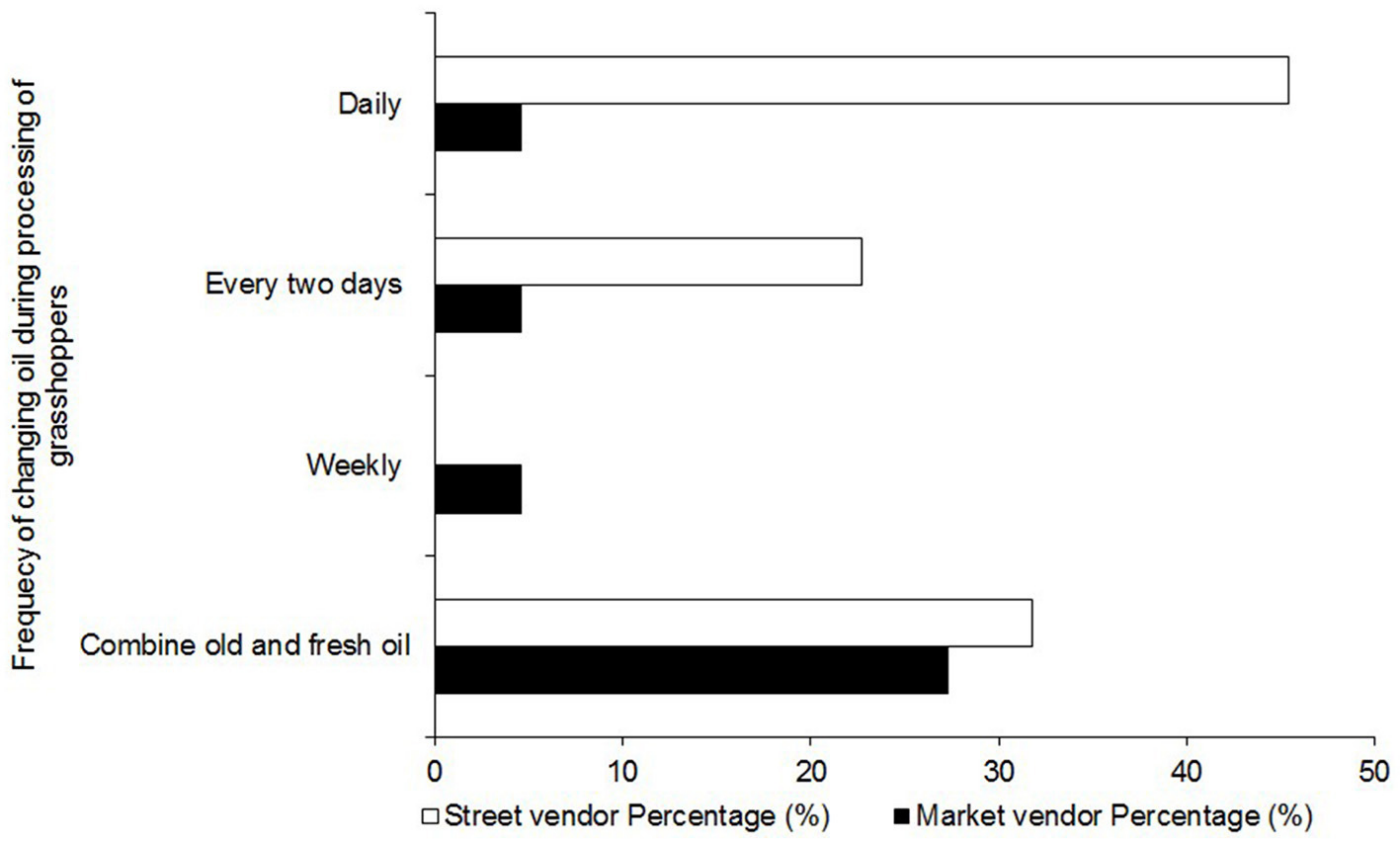
Frequency of use of oil in deep frying *nsenene* (grasshopper).

The quality of oil used for deep frying *nsenene* influences their safety and quality. In this study, respondents were asked how often they change their deep-frying oil as a proxy for the quality of the oil. While the majority of the market vendors (66.7%) never changed the oil but topped up old oil with fresh oil, some street vendors (45.5%) reported that they use fresh oil for deep frying daily (Figure 11). This result probably may be attributed to low volumes of grasshoppers handled by street vendors relative to their market counterparts. From a nutritional standpoint, repeatedly using the same deep frying oil, causes a chain of oxidative reactions that lead to the formation of free radicals, acrylamides, and trans-fats which cause cancers and cardiovascular diseases thus putting consumers at risk (Goswami et al., 2015).

#### 3.8.4 Vendors' preference for shelf life and preservation of processed and unprocessed grasshoppers

Due to a lack of legislation in the *nsenene* subsector, there are currently no standards that give the maximum shelf life of processed and unprocessed grasshoppers. Vendors were therefore asked what they perceive to be the length of time that unprocessed and processed grasshoppers remain fit for processing and consumption, respectively. About 50% of both street and market vendors agreed that raw *nsenene* remains fresh only for 1 day after which almost all insects die and begin to decompose (Table 2). The two categories of vendors however reported different perceptions of the shelf life of deep-fried *nsenene*. About 50% of market vendors concluded that deep-fried *nsenene* remained fit for sale and consumption for 60 days, whereas street vendors argued that they can stay fit for human consumption even up to 90 days; beyond which they develop undesirable properties such as off-odors. It was observed that unprocessed grasshoppers are usually stored spread out on a sack on the ground or on a raised surface which is shaded and airy. According to the vendors the cool environment reduces the rate of death and decomposition before *nsenene* is sold or processed. Deep-fried grasshoppers are generally stored in opaque carton boxes at ambient temperatures for long-term storage while awaiting sale. During the sale of *nsenene*, it was observed that deep-fried ones are deliberately exposed on a tray to be seen by buyers, a practice which exposes them to contamination from the environment. It was common practice to have street vendors keep the *nsenene* on charcoal warmers placed on push carts, a practice that keeps them at danger zone temperatures (5–60°C) enabling rapid growth of bacteria (Maciel et al., 2012). The respondents were also asked about the use of refrigeration as a potential strategy to preserve *nsenene*. Although studies such as Ssepuuya et al. (2016) show that refrigeration can reduce the rate of spoilage and extend the shelf life of *nsenene*, only 16% of market vendors and only 5% of street vendors practice refrigeration. Vendors believe that refrigeration reduces the quality of the raw grasshoppers making the insects watery and mushy. This could be due to thawing damage which occurs when food is frozen and thawed repeatedly (Archer, 2004).

**Table 2:** Vendors' knowledge on shelf life of processed and unprocessed edible grasshoppers.

**Edible grasshopper products shelf life (days)**				
	**Market vendors**		**Street vendors**	
	**Mean**	**Median**	**Mean**	**Median**
Harvested raw grasshoppers	1.01	1	1.08	1
Deep fried grasshoppers	77.9	60	158	90

### 3.9 Hygiene and sanitation practices of grasshopper vendors

**Figure 12:**
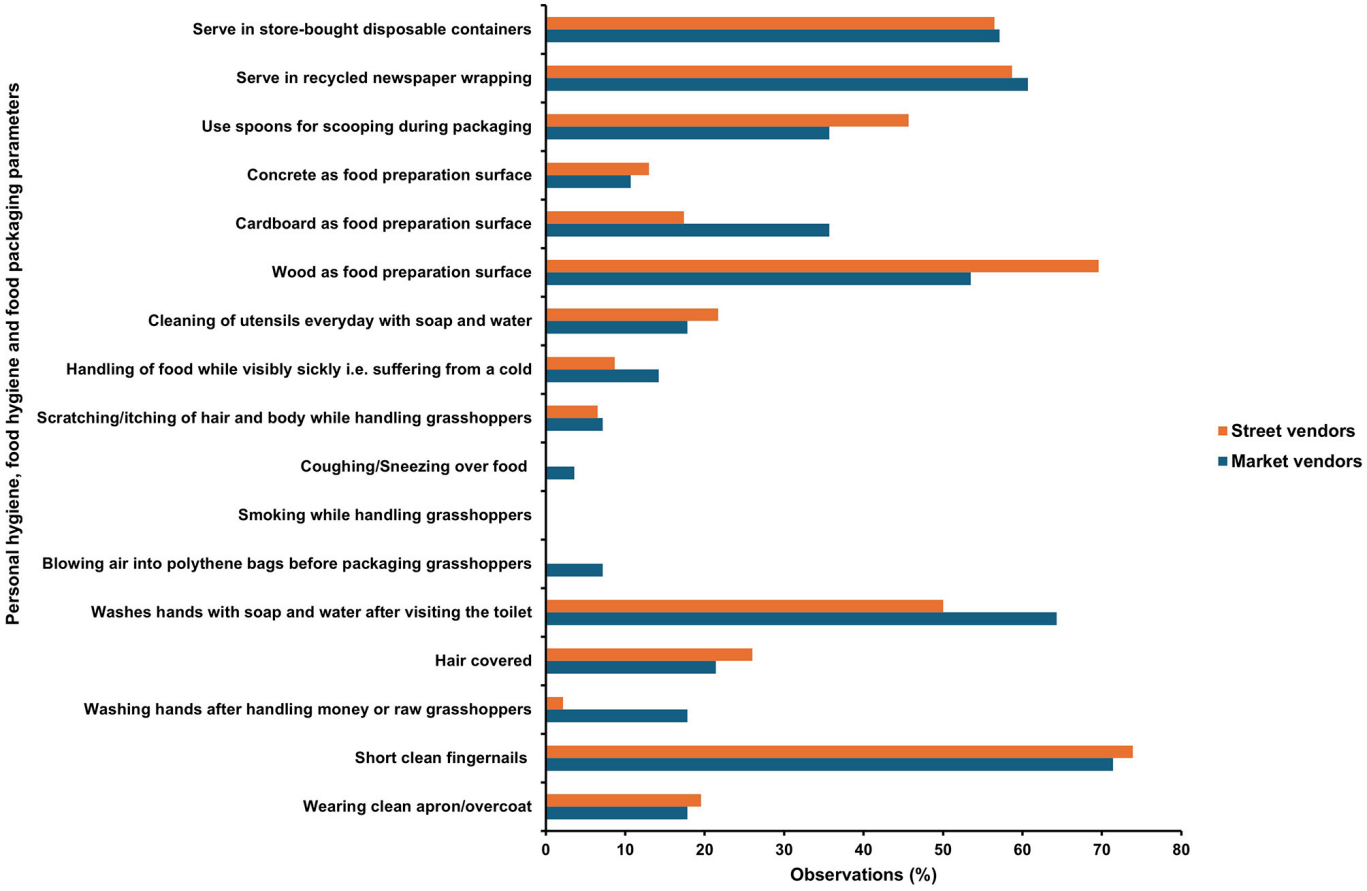
Prevailing personal and food hygiene practices of *nsenene* (grasshopper) vendors.

In our study, the hygiene and sanitation practices of vendors were observed against a checklist. Edible grasshopper vendors surveyed had poor knowledge of personal hygiene (Figure 12). Market vendors had a mean score of 48% which was slightly lower than the mean score for the street vendors of 52%. Similarly, Baş et al. (2006) observed a low food safety knowledge score of 43.4% among Turkish street vendors. Some of the positive observations made were most of the vendors (71.4% MV and 73.9% SV) kept their fingernails clean and short during food handling (Figure 12). Vendors were observed not to sneeze or cough over food and did not blow air into the packaging bags to open them. However, many vendors did not exhibit proper hand washing during food handling. Washing of hands every time they got contaminated was practiced by only 18% of MV and 2% of SV. About 64% of MV and 50% of SV reported that they washed their hands with soap and water after using the toilet. During packaging, it was a common practice among 35% of MV and 45% of SV to use spoons to package processed grasshoppers, while they used their bare hands to package the unprocessed ones. This poses a risk of cross-contamination from raw to cooked insects. In the case of food hygiene practices, we learned that it was not common practice to wash the utensils used in the nsenene trade daily. Only 17.1% of MV and 21.7% of SV washed their utensils every day. However, it was not apparent, from our study how well utensils were washed and whether they used soap and hot water since grasshoppers are a greasy food. Failure to wash storage containers increases the probability of microbial contamination, posing a risk of foodborne pathogens in the food.

## 4 Conclusion

The microbial contaminant levels reported in the present study are considerably high and above acceptable limits for ready-to-eat food products with great potential to cause human illnesses (Food Safety Authority of Ireland, 2019). The presence of pathogenic bacteria and fungi also implies the great potential of *nsenene* to cause food-borne illnesses in consumers. Our study also revealed that although, women below the age of 35 years dominated the business space of “*nsenene*,” they had very low levels of education. Thus, both street and market vendors had low knowledge in areas related to food safety (i.e., personal and food hygiene practices). Market and street vendors prepared and sold processed grasshoppers in unhygienic conditions that could create an enabling environment for pathogens to grow and multiply to levels or produce toxins that could be harmful to consumers. This is because no approved processing, handling, repacking, or storage technologies exist for what constitutes “ready-to-eat” grasshopper products. Therefore, decision-making authorities involved in food protection should take urgent measures to address some of these issues by developing protocols to assess the safety of specific types of processed grasshoppers held at ambient temperature. The sector also lacks regulations such as the provision of food handlers with medical food handlers' certificates which would be accompanied by food safety and hygiene training. Therefore, there is an urgent need to adopt regulations and guidelines on good hygiene practices in the production of *nsenene* to address potential food safety concerns along the value chain.

## Data availability statement

The original contributions presented in the study are included in the article/Supplementary material, further inquiries can be directed to the corresponding author.

## Author contributions

LM-K: Conceptualization, Data curation, Formal analysis, Investigation, Methodology, Validation, Writing – original draft, Writing – review & editing, Visualization. JI: Conceptualization, Validation, Writing – review & editing, Supervision. LN: Conceptualization, Validation, Writing – review & editing, Supervision. KA: Validation, Writing – review & editing, Conceptualization. FK: Methodology, Validation, Writing – review & editing, Investigation, Visualization. FO: Conceptualization, Formal analysis, Investigation, Methodology, Validation, Visualization, Writing – review & editing. GD: Conceptualization, Data curation, Formal analysis, Investigation, Methodology, Validation, Visualization, Writing – review & editing. TC: Conceptualization, Data curation, Formal analysis, Investigation, Methodology, Project administration, Resources, Supervision, Validation, Writing – original draft, Writing – review & editing. SS: Conceptualization, Data curation, Funding acquisition, Methodology, Project administration, Resources, Supervision, Validation, Writing – original draft, Writing – review & editing.
