# Supplementary material for: Vendors' handling practices of edible long-horned grasshoppers (Ruspolia differens) products and implications on microbial safety
Source: Front Microbiol. 2024 May 6;15:1385433. doi: 10.3389/fmicb.2024.1385433 (PMC11102984; doi:10.3389/fmicb.2024.1385433)
Supplement: Supplementary file 1 [file Data_Sheet_1.pdf]

Supplementary S1

Food Safety Knowledge and Hygiene Practices Questionnaire

General Information

Questionnaire no……………. Vendor number………………..Collector number………………. Date……………………

Market name……………………………………Town/City……………………………...

Section A: Socio-demographic Characteristics

1. Gender M [ ] F [ ]

2. Age 18-25 [ ] 26-25 [ ] 36-55 [ ] Above 55 [ ]

3. Highest level of education attained

No education [ ] Primary [ ] Secondary [ ] Tertiary [ ]

4. Marital Status

Never Married [ ] Married monogamous [ ] Married Polygamous [ ] Divorced/Separated[ ] Widowed [ ]

5. Have you ever received any training on food safety, hygiene and sanitation?

Yes [ ] No [ ]

6. Length of time in the job/ business

Less than 1 year [ ] 1-5yrs [ ] 6-10yrs [ ] 10-15yrs [ ] 16-20yrs [ ] above 20yrs [ ]

Section B: Food safety Knowledge

I. Grasshopper processing

1. What type of vendor are you?

Market vendor [ ] Street Vendor [ ]

2. Where do you prepare your *nsenene* from

Market stall [ ] Home [ ] Other (Specify)…………………….

3. What type of processed grasshopper do you specialize in

Fresh [ ] Boiled [ ] Fried without oil [ ] Deep fried [ ]

4. What do you consider when buying *nsenene* from the collectors

Freshness [ ] Price [ ] Type of packaging [ ] Cleanliness of collector/Transport Vehicle [ ]

5. Do you think *nsenene* is clean when you receive it from the collector or does it need sorting and cleaning

   Clean [ ] Requires Sorting/ Cleaning [ ]

6. Which parts are removed during the cleaning/ sorting process

Wing [ ] Hind legs [ ] Others (specify)……………………………

7. Why is it important to remove these parts/ Why are they not eaten?

Can cause injury to consumer [ ] Not liked by consumer [ ] Other (specify)……………………...

8. Are the insects washed with water before cooking

Yes [ ] No [ ]

9. How many times do the insects need to be washed in order to be completely clean

Once [ ] Twice [ ] repeatedly until water comes out clear [ ]

10. What is your source of water supply?

Public tap [ ] Bring from home [ ] Buy from water vendors [ ] Private property i.e. Church [ ]

11. During cooking, how long are the grasshoppers,

a) Boiled ……………………

b) Deep-fried…………………….

c) Pan-fried without oil……………………………..

12. How often do you change the oil

Daily [ ]   Weekly [ ]   Monthly [ ]   other (specify).............

13. How long can cooked *nsenene* stay without getting spoilt without refrigeration

Less than 24hrs [ ] 24hrs [ ] 24-36hrs [ ] 36-48hrs [ ] More than 48 hrs [ ]

14. How long does the fresh *nsenene* last without getting spoilt without refrigeration

Less than 24hrs [ ] 24hrs [ ] 24-36hrs [ ] 36-48hrs [ ] More than 48 hrs [ ]

15. How is the food stored while vending

Open air [ ]   In covered containers [ ]   Cooler box/refrigerator [ ]

16. Is there a chance of mixing fresh and processed grasshoppers

Yes [ ]   No [ ]

17. Type of surface used by the vendor for preparing food

Wood [ ] Cardboard [ ] Aluminum [ ] Concrete [ ] Other (specify)…………

18. Type of packaging used to sell *nsenene*

Plastic paper [ ]   Newspaper wrapping [ ]   Kraft Paper(brown) [ ] Other (Specify)………………….

19. How do you handle leftover *nsenene*

Throw away [ ] Store for selling next day [ ] Take home to eat [ ] No leftovers [ ]

20. How are leftovers stored

Room temperature [ ]   Refrigerator [ ]

21. If stored to be sold later, are they?

Sold first as they are [ ] Mixed with new stock [ ] Re-cooked and sold separately [ ]

II. Food Borne Illnesses

1. Do you have a valid food handlers' certificate?

Yes [ ] No [ ]

2. Have you ever suffered any kind of illness after consuming *nsesene*

Yes [ ] No [ ]

3. Have any of your customers ever complained of falling sick after consuming *nsenene*

Yes [ ] No [ ]

4. How would you describe the illness suffered (By either you or the customer)

…………………………………………………………………………………………………

…………………………………………………………………………………………………

…………

5. Did you or the customer suffer any of the following symptoms

Diarrhea [ ] Vomiting [ ] Stomach Pain [ ] Itchy skin/ Rashes [ ] Difficulty breathing [ ]

Swollen face/lips [ ]

Section C: Hygiene and sanitation

I. Toilet Facilities

1. Access/use of toilet

Yes [ ] No [ ]

2. Toilet used

Public toilet [ ] Private property i.e. church [ ] Street [ ]

3. Hand washing after toilet

Running tap water within the facility [ ] Water in a container within facility [ ]

Water in container at the vendors selling point [ ]

Is there soap where they wash their hands

Yes [ ] No [ ]

II. Personal Hygiene

The interviewer will tick what they observe in the checklist below

Use a tick ✓ for YES and an X for No

Talking while working with food	
Handling food and money without washing hands in between	
Dry sweat in a way that can contaminate food	
Handles *nsenene* without gloves / any hand covering	
Vendor is wearing a clean apron or overcoat	
Vendor has short clean finger nails	
Smoking while handling the insects	
Vendor’s hands are clean	
He/she is not wearing any jewelry	
His/her hair is covered	
Blows air into polythene bag before use	
Vendor is coughing/sneezing over food	
He/she is itching hair/body while working with food	
Vendor looks like he/she is suffering from a cold while working	
